# Structural mechanism of bivalent histone H3K4me3K9me3 recognition by the Spindlin1/C11orf84 complex in rRNA transcription activation

**DOI:** 10.1038/s41467-021-21236-x

**Published:** 2021-02-11

**Authors:** Yongming Du, Yinxia Yan, Si Xie, Hao Huang, Xin Wang, Ray Kit Ng, Ming-Ming Zhou, Chengmin Qian

**Affiliations:** 1 School of Biomedical Sciences, The University of Hong Kong, Hong Kong Island, Hong Kong; 2 Department of Biomedical Sciences, The City University of Hong Kong, Kowloon, Hong Kong; 3 Department of Pharmacological Sciences, Icahn School of Medicine at Mount Sinai, New York, NY USA

**Keywords:** Epigenetics, X-ray crystallography

## Abstract

Spindlin1 is a unique multivalent epigenetic reader that facilitates ribosomal RNA transcription. In this study, we provide molecular and structural basis by which Spindlin1 acts in complex with C11orf84 to preferentially recognize non-canonical bivalent mark of trimethylated lysine 4 and lysine 9 present on the same histone H3 tail (H3K4me3K9me3). We demonstrate that C11orf84 binding stabilizes Spindlin1 and enhances its association with bivalent H3K4me3K9me3 mark. The functional analysis suggests that Spindlin1/C11orf84 complex can displace HP1 proteins from H3K4me3K9me3-enriched rDNA loci, thereby facilitating the conversion of these poised rDNA repeats from the repressed state to the active conformation, and the consequent recruitment of RNA Polymerase I for rRNA transcription. Our study uncovers a previously unappreciated mechanism of bivalent H3K4me3K9me3 recognition by Spindlin1/C11orf84 complex required for activation of rRNA transcription.

## Abstract (web summary)

Spindlin1 is an epigenetic reader that facilitates ribosomal RNA transcription. Here the authors reveal in vitro and structural evidence suggesting that Spindlin1 acts together with C11orf84 to recognize noncanonical bivalent mark of trimethylated lysine 4 and lysine 9 present on histone H3 tail (H3K4me3K9me3).

## Introduction

Transcription of ribosomal DNA, the first key step of ribosome biogenesis, is tightly regulated by multi-layer epigenetic mechanisms involving DNA methylations, histone modifications, chromatin remodeling, and non-coding RNA regulation^1,2^. Although ribosomal RNA production represents the most active transcription, only a subset of rRNA genes is actively transcribed at a given time, and the inactive rRNA genes exist in the compact heterochromatic state that is typically associated with repressive histone marks such as methylated histone H3 lysine 9 (H3K9), H3K27, and H4K20. Intriguingly, rDNA chromatin is metastable, and can readily respond to environmental stimuli and developmental cues. Dynamic changes of epigenetic modifications can help shift the balance between repressed and active rRNA genes. For instance, chromatin remodeling complex NoRC recruits corepressors such as SETDB1 and HDAC1/2 to rDNA promoters to establish trimethylated H3K9 (H3K9me3) for rRNA gene silencing^3^. On the other hand, overexpression of the H3K9 demethylase PHF8 facilitates rRNA transcription^4^, and the H3K4me3 demethylase KDM2B was found to localize in the nucleolus and repress rRNA transcription^5^.

To meet the increasing demand for ribosomes in fast growing cells, a higher level of rRNA transcription is achieved through the more copies of actively transcribed rRNA genes and the higher transcription rate. To convert the rRNA genes in repressed and closed conformation into the open and transcription-permissive state, methylation on H3K4 and demethylation on H3K9 at the ribosomal RNA gene promoter region needs to be established. Although H3K4me3 and H3K9me3 are generally perceived to be mutually exclusive, bivalent H3K4me3 and H3K9me3 chromatin domains have been reported in different cell types^6–9^. Interestingly, it was reported that binding of KDM4A and KDM4C to H3K4me3 through their double Tudor domains greatly facilitates these enzymes to efficiently demethylate H3K9me3^10,11^, suggesting the coexistence of H3K4me3 and H3K9me3 on the same histone H3 and likely a functional crosstalk between these two seemingly opposing epigenetic marks. It has been suggested that some euchromatic rRNA genes may exist in a poised state harboring canonical bivalent mark H3K4me3K27me3^6,12^, or non-canonical bivalent mark H3K4me3K9me3^13,14^, thus allowing their timely activation in response to environmental changes. However, our current mechanistic understanding of how bivalent histone marks function with respect to corresponding singular histone marks is very limited. In this study, we provided new structural and functional insights into a previously unappreciated mechanism by which Spindlin1 in complex with C11orf84 recognizes K4me3 and K9me3 dual marks on the same histone H3 tail. This unique bivalent histone recognition results in dissociation of HP1 from condensed rDNA chromatin to relax the chromatin structure and facilitate the recruitment of RNA Polymerase I, leading to productive transcription of rRNA.

## Results

### Non-canonical bivalent H3K4me3K9me3 recognition by Spindlin1/C11orf84 complex

**Fig. 1:**
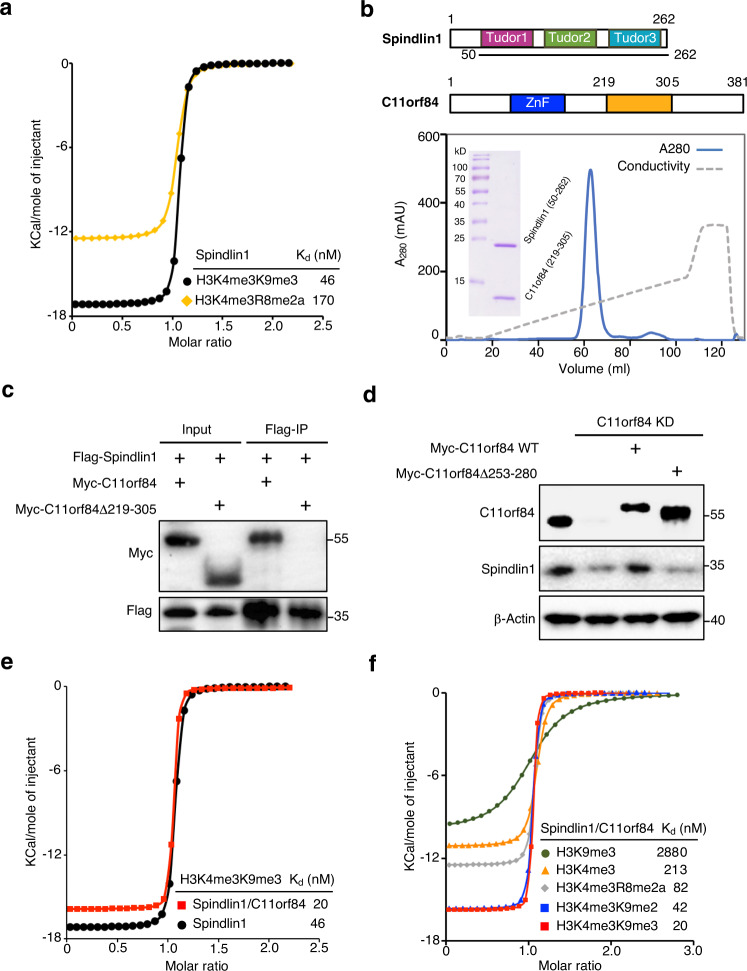
Spindlin1/C11orf84 complex prefers to recognize non-canonical bivalent mark H3K4me3K9me3. **a** Quantitative ITC measurements suggest that Spindlin1 binds H3K4me3K9me3 dual mark with a fourfold increase in binding affinity over H3K4me3R8me2a. The insert lists the calculated dissociation constant (*K*_d_). **b** The top panel shows protein architecture of Spindlin1 and C11orf84. The lower panel shows that Spindlin1 (aa: 50–262) forms a stable complex with C11orf84 (aa: 219–305) as analyzed by anion-exchange chromatography and SDS-PAGE gel of the peak fraction. **c** Coimmunoprecipitation (Co-IP) shows the region (aa: 219–305) of C11orf84 is responsible for Spindlin1 binding. **d** Immunoblotting shows the Spindlin1 protein level is dramatically reduced in C11orf84 stable knockdown (C11orf84 KD) U2OS cells, and ectopic expression of wild-type C11orf84 but not the C11orf84Δ253-280 mutant restored the Spindlin1 protein level. **e** ITC measurement shows the Spindlin1/C11orf84 heterodimer binds twofold stronger to H3K4me3K9me3 peptide than Spindlin1 alone. **f** ITC fitting curves for binding of Spindlin1/C11orf84 complex to various H3 peptides with dissociation constant (*K*_d_) values indicated. The thermodynamic parameters are listed in Supplementary Table 1. Source data are provided as a Source Data file.

Human Spindlin1 was initially characterized as a reader of H3K4me3 and recently reported to recognize H3K4me3R8me2a dual mark^15,16^. Given that Spindlin1 facilitates ribosomal RNA transcription^15,17^, we explored whether Spindlin1 binds to H3K4me3K9me3 at poised rRNA genes. We first carried out quantitative ITC measurement to show that Spindlin1 binds to H3K4me3K9me3 with a *K*_d_ of 46 nM, about fourfold higher than that to H3K4me3R8me2a (Fig. 1a and Supplementary Fig. 1a).

Because C11orf84 was recently reported to bind Spindlin1 and cause it to dissociate from the chromatin^18^, we investigated and found that full-length C11orf84 and Spindlin1 (aa: 50–262) form a stable heterodimer as shown by analytical gel filtration (Supplementary Fig. 1b). We mapped the region spanning residues 219–305 in C11orf84 responsible for Spindlin1 binding using limited proteolysis-coupled mass spectrometry. The result was confirmed by observation of a stable protein complex of Spindlin1 (aa: 50–262) and C11orf84 (aa: 219–305) in anion-exchange chromatography (Fig. 1b), and by coimmunoprecipitation (co-IP) assay showing that C11orf84Δ219-305 truncation mutant lost Spindlin1 binding (Fig. 1c). Furthermore, we found that the protein level of Spindlin1 is substantially reduced in C11orf84 knockdown U2OS cells, and ectopic expression of wild-type C11orf84 but not the C11orf84Δ253-280 mutant can restore the Spindlin1 protein level (Fig. 1d). These results suggested that C11orf84 is likely a bona fide binding partner of Spindlin1 and can stabilize Spindlin1 through the direct interaction.

In contrast to the previous report^18^, we showed that overexpression of C11orf84 in HEK293T cells does not block the binding of Spindlin1 to methylated histone H3 (Supplementary Fig. 1c). Our observation is consistent with a previous proteomic-based study that C11orf84 can be pulled out from H3K4me3 peptide^19^, and also in good agreement with a more recent study that a Spindlin1 inhibitor bound on H3K4me3 pocket of Tudor 2 can pull down both Spindlin1 and C11orf84^20^. Moreover, we performed ITC measurement to reveal that Spindlin1/C11orf84 heterodimer binds to H3K4me3K9me3 peptide with a *K*_d_ of 20 nM, about twofold higher than that of Spindlin1 alone, suggesting that C11orf84 enhanced Spindlin1 binding to H3K4me3K9me3 peptide (Fig. 1e). Interestingly, Spindlin1/C11orf84 complex also showed the strong binding to H3K4me3K9me2 which is a preferred substrate for PHF8 (Fig. 1f)^4^. ITC data also suggested that H3K4me3 is a primary mark responsible for the binding, the addition of H3K9me2/3 mark significantly enhanced the binding, indicative of a multivalent readout of dual H3K4me3K9me2/3 mark by the Spindlin1/C11orf84 complex (Supplementary Table 1). It is also worth mentioning that the interaction of Spindlin1/C11orf84 with H3K4me3K9me3 represents one of the strongest bindings in methylated histone H3 recognition reported so far^21^, provided that most reported methyl histone binding is in the micromolar or sub-micromolar range.

### Crystal structure of Spindlin1/C11orf84 complex bound to H3K4me3K9me3

**Fig. 2:**
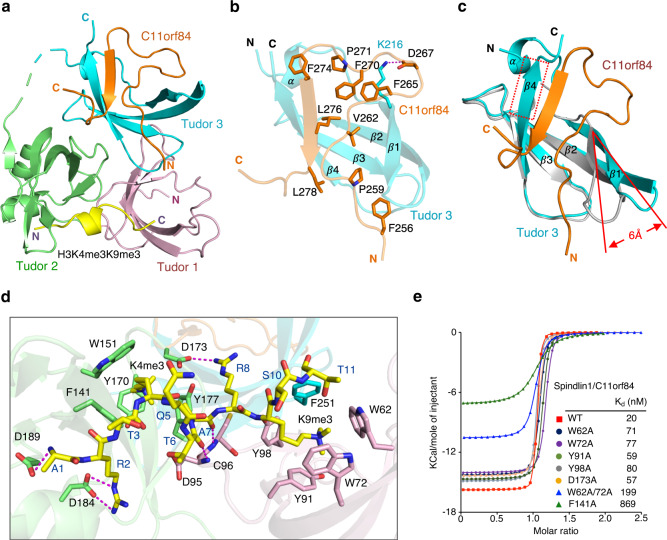
Structural details of Spindlin1/C11orf84/H3K4me3K9me3 ternary complex. **a** Cartoon representation of the structure of Spindlin1/C11orf84 bound to H3K4me3K9me3 peptide. Spindlin1 Tudor 1, Tudor 2, and Tudor 3 domains are colored in light pink, green, and cyan, respectively. The C11orf84 segment and H3K4me3K9me3 peptide are colored in orange and yellow, respectively. **b** Detailed view of the interaction between C11orf84 and Spindlin1 Tudor 3 domain. Key residues involved in the interaction are depicted as stick models and labeled. Hydrogen bonds and salt bridges are shown in magenta dashed lines. **c** Structural comparison with the previous determined Spindlin1/H3K4me3R8me2a binary complex (PDB: 4MZF, colored in light gray) revealed that binding of C11orf84 fragment induced conformational changes in Spindlin1 Tudor 3 domain. The red dash box highlights the Y256-K260 segment of Tudor 3 adopts a β-strand conformation upon C11orf84 binding. In addition, the Tudor 3 β1–loop–β2 region was pushed outward with ~6 Å in distance to accommodate the C11orf84 fragment. **d** The detailed interaction network of Spindlin1/C11orf84 with H3K4me3K9me3 peptide. The H3K4me3K9me3 segment is depicted as yellow sticks. Hydrogen bonds and salt bridges are shown in magenta dashes. **e** ITC fitting curves for binding of Spindlin1/C11orf84 complex including Spindlin1 wild type and mutants to the H3K4me3K9me3 peptide, along with the calculated *K*_d_. The thermodynamic parameters are listed in Supplementary Table 3.

To gain mechanistic insight into the bivalent H3K4me3K9me3 recognition, we determined the crystal structure of Spindlin1/C11orf84/H3K4me3K9me3 ternary complex at 1.60 Å resolution (crystallographic statistics is given in Supplementary Table 2). The ternary structure revealed that methyl histone peptide lies across Tudor 1 and 2 domains, whereas the C11orf84 fragment associates with Tudor 3 domain (Fig. 2a and Supplementary Fig. 2a).

The electron density map of C11orf84 amino-acid sequence T255-Q283 is clearly defined and adopts a unique stem–loop conformation (Supplementary Fig. 2b). This segment inserts into the cleft between the strand β1 and β4 of Spindlin1 Tudor 3 to form a closed β barrel-like structure (Fig. 2b). Structural comparison with the previous determined Spindlin1/H3K4me3R8me2a structure (PDB: 4MZF) showed that binding to C11orf84 caused obvious conformational changes in Tudor 3 domain: (i) Y256-K260 segment of Spindlin1 Tudor 3 adopts a β-strand conformation and forms an antiparallel β-sheet with the β-strand adopted by the F274-L278 segment of C11orf84; (ii) part of Tudor 3 β1–loop–β2 region is pushed outward with ~6 Å in distance to accommodate the C11orf84 fragment (Fig. 2c and Supplementary Fig. 2c). In addition, the binding to C11orf84 appears to stabilize Spindlin1 structure as the N-terminal A204-D211 region including an α helix of Tudor 3 domain is well-defined but was invisible in the previous determined Spindlin1 structures^15,16^.

The H3K4me3K9me3 peptide fits snugly on the surface of Tudor 1 and 2 of Spindlin1 (Fig. 2d and Supplementary Fig. 3a, b). Simultaneous recognition of K4me3 and K9me3 appears to be facilitated by the distance adjustment through the short α-helix formation of the H3 fragments consisting of residues 4–7. This is distinct from the extended conformation of H3 peptide observed in Spindlin1/H3K4me3 and Spindlin1/H3K4me3R8me2a structures^15,16^. Trimethylated K4 is inserted into the aromatic pocket formed by F141, W151, Y170, and Y177 of Tudor 2 domain whereas trimethylated K9 is recognized by an aromatic cage formed by residues W62, W72, Y91 and Y98 of Tudor 1, and F251 of Tudor 3 domain (Supplementary Fig. 3c, d). In addition to cation-π and methyl-π interactions induced by dual methylated lysine recognition, the N-terminal amino group of H3A1 forms a hydrogen bond with the side-chain carboxylate group of D189, while the guanidino moiety of H3R2 is ion-paired with the side-chain carboxylate group of D184. The backbone carbonyl groups H3T6 and H3A7 form a hydrogen bond with backbone amide groups of D95 and C96 of Tudor 1, respectively. Furthermore, the H3R8 side-chain guanidino group forms a hydrogen bond with the side-chain carboxylate group of D173 of Tudor 2.

To corroborate the significance of specific interactions identified in the structure of the Spindlin1/C11orf84/H3K4me3K9me3 complex, we generated a number of Spindlin1 mutants and quantitatively evaluated the effects of mutation by ITC measurement (Fig. 2e and Supplementary Table 3). Single mutation of W62, W72, Y91, or Y98 with alanine led to about threefold reduction in binding affinity of H3K4me3K9me3 peptide, W62A/W72A double mutation caused about 10-fold loss in binding affinity, whereas single mutation of F141A resulted in an approximately 40-fold binding loss, confirming that H3K4me3 is the primary mark responsible for the binding. We also performed co-IP assay to validate the structural findings. Indeed, C11orf84Δ253-280 truncated mutant does not show any interaction with Spindlin1 (Supplementary Fig. 3e). Co-IP assay has also confirmed that C11orf84 is able to interact with other Spindlin family proteins (Supplementary Fig. 4a–d). This is expected given the fact that the sequence of Tudor 3 in all five Spindlin family proteins are highly conserved (Supplementary Fig. 4e). Interestingly, the mRNA expression level of Spindlin1 is much higher than other four Spindlin family members in both U2OS and HEK293T cells although all Spindlin members can interact with H3K4me3K9me3 and localize in the nucleolus (Supplementary Fig. 5). It is worth mentioning that human *SPIN*1 resides on chromosome 9 while other four *SPIN* genes are located close to the pericentromeric region of X chromosome^22^.

### Spindlin1 binding to C11orf84 and H3K4me3K9me3 stimulates rRNA transcription

**Fig. 3:**
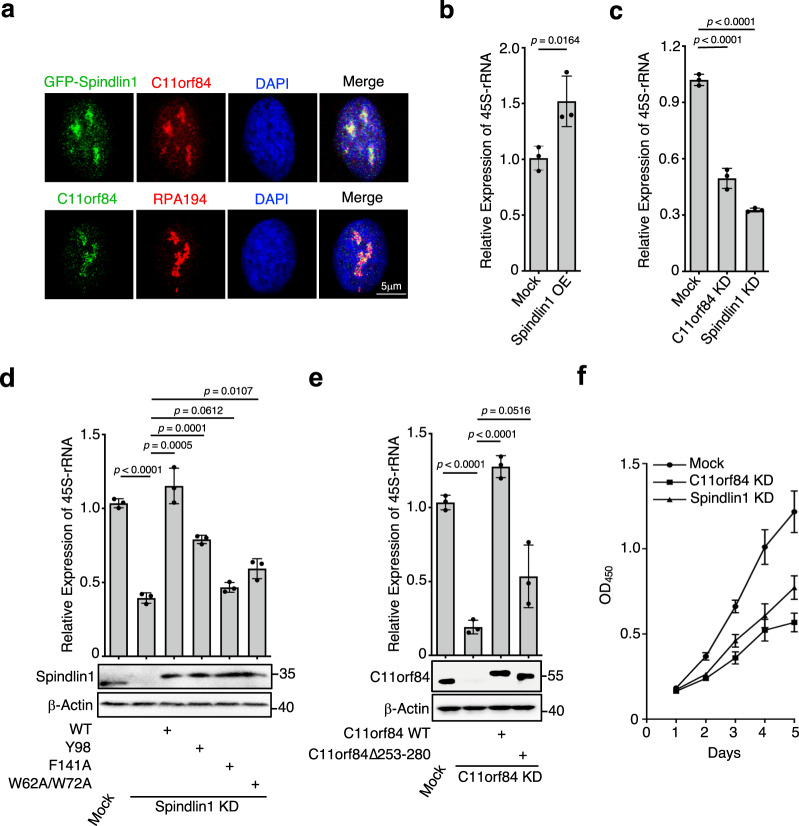
Binding to C11orf84 and H3K4me3K9me3 is required for Spindlin1 to stimulate rRNA transcription. **a** C11orf84 colocalizes with Spindlin1 and RPA194 in the nucleolus. C11orf84 and RPA194 were visualized by indirect immunofluorescence whereas GFP-Spindlin1 by direct fluorescence. Nuclei were stained with DAPI. Scale bar: 5 μm. **b** Overexpression of Spindlin1 stimulates rRNA transcription. The 45S pre-rRNA level was determined by RT-qPCR in HEK293T mock cells and Spindlin1-overexpressed (Spindlin1 OE) cells. **c** RT-qPCR reveals that depletion of Spindlin1 or C11orf84 results in a reduced level of 45S pre-rRNA transcript in U2OS cells. **d** Ectopic expression of wild-type Spindlin1 but not the mutated forms that impaired the binding to either K4me3 or K9me3 of histone H3 restored the rRNA transcription level in Spindlin1 stable knockdown U2OS (Spindlin1 KD) cells. RT-qPCR was performed to determine the level of 45S pre-rRNA. Western blot shows the level of Myc-tagged wild type and mutant Spindlin1. **e** Ectopic expression of wild-type C11orf84 but not the truncated form that impaired the binding to Spindlin1 restored rRNA synthesis in C11orf84 stable knockdown U2OS (C11orf84 KD) cells. **b**–**e** Data are shown as mean ± SD (*n* = 3 independent repeated experiments). The *p* values were calculated by two-tailed unpaired *t*-test. **f** Knockdown of Spindlin1 or C11orf84 inhibits cell proliferation. Cell number was counted by Cell Counting Kit-8 (CCK-8) on a daily basis for a period of 5 days. Data are presented as mean ± SD (*n* = 5 independent experiments). The *p* values were calculated with two-way ANOVA test followed by Dunnett’s multiple comparisons test. Spindlin1 KD versus Mock, *p* values are 0.0036, 0.0005, 0.0045, and 0.004, respectively from day 2 to day 5; C11orf84 KD versus Mock, *p* values are 0.0019, 0.0004, 0.0004, and <0.0001, respectively, from day 2 to day 5. Source data are provided as a Source Data file.

The previous study indicated Spindlin1 is enriched in the nucleolus and particularly bound to active rDNA loci^17^. Since Spindlin1 and C11orf84 form a stable complex both in vitro and in vivo, we performed co-immunostaining and confirmed that C11orf84 accumulates in the nucleolus and colocalizes with Spindlin1 as well as RPA194 (the largest subunit of RNA Pol I), suggesting that C11orf84 is enriched in the active rDNA sites (Fig. 3a). To evaluate how Spindlin1 and C11orf84 regulate ribosomal RNA transcription, we measured 45S pre-rRNA level by RT-qPCR in Spindlin1-overexpressed cells as well as in Spindlin1 or C11orf84 stable knockdown cells (Fig. 3b, c). Overexpressing Spindlin1 stimulates rRNA transcription in HEK293T cells, whereas depletion of either Spindlin1 or C11orf84 leads to a significant reduction in the 45S pre-rRNA level. Notably, ectopic expression of wild-type Spindlin1 but not F141A single mutant or W62A/W72A double mutant in Spindlin1 knockdown cells restored the level of 45S pre-rRNA (Fig. 3d), suggesting the binding to a dual methyl mark H3K4me3K9me3 is required for Spindlin1/C11orf84 to effectively activate rRNA transcription. In addition, ectopic expression of wild type but not C11orf84Δ253-280 truncated mutant can efficiently enhance rRNA transcription in C11orf84 stable knockdown cells (Fig. 3e). As ribosome biogenesis drives cell proliferation and growth, we performed cell counting assay to show a decreased cell proliferation in Spindlin1- or C11orf84-depleted cells (Fig. 3f).

### Spindlin1/C11orf84 complex displaces HP1 from H3K4me3K9me3-enriched rDNA loci

The majority of H3K9me2/3 marks in constitutive and temporary silenced rRNA genes is associated with HP1 proteins which includes three isoforms HP1α, HP1β, and HP1γ^23,24^. HP1 N-terminal chromodomain recognizes H3K9me3 and C-terminal chromo shadow domain dimerizes to bridge neighboring nucleosomes for compact chromatin structure formation. Previous structural analysis suggested that trimethylation on H3K4 has no impact on HP1 chromodomain binding to H3K9me2/3^25,26^. On the other hand, binding of KDM4A to H3K4me3 through its C-terminal double Tudor domain contributes to the catalysis of H3K9me3 demethylation and rRNA transcription activation^10,13^. The binding to H3K4me3 via its N-terminal PHD finger stimulates PHF8 demethylase activity towards dimethylated H3K9, and consequently activates the transcription of rRNA genes^4,27^. Intriguingly, the binding affinity of KDM4A or PHF8 with H3K4me3K9me2/3 is very close to that of HP1^11,28^, which means introducing trimethylation on H3K4 does not readily dissociate HP1 from H3K9me2/3-enriched nucleosomes to expose H3K9me2/3 for KDM4A or PHF8 to remove methyl marks^4,13,27^. The strong binding of Spindlin1/C11orf84 complex to H3K4me3K9me2/3 dual marks prompted us to investigate if the Spindlin1/C11orf84 complex can displace HP1 proteins from H3K4me3K9me2/3-poised rDNA chromatin.

**Fig. 4:**
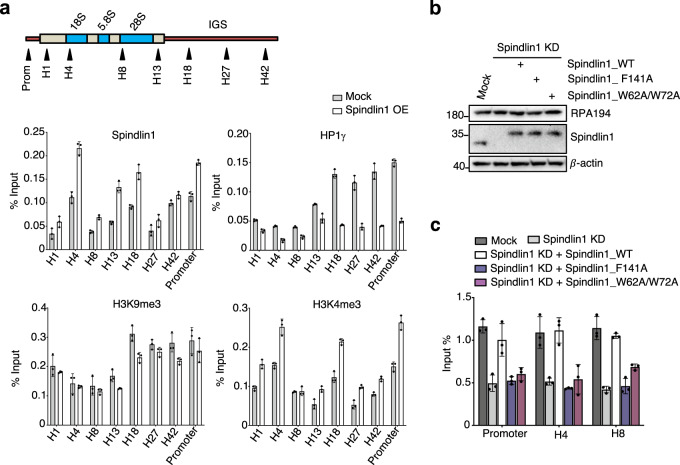
Spindlin1/C11orf84 complex displaces HP1γ from the rDNA foci enriched with H3K4me3K9me3 bivalent mark and facilitates the recruitment of RNA Pol I. **a** The top panel is a schematic diagram of a rDNA repeat. Arrows indicate the positions of eight primer pairs designed for qPCR analysis of immunoprecipitated DNA. The lower four panels are ChIP-qPCR measurement of the relative occupancy of Spindlin1, HP1γ, H3K9me3, and H3K4me3 on different regions of rDNA in HEK293T cells expressing Myc-tagged Spindlin1 or an empty vector (Mock). The precipitated DNA was analyzed by qPCR using primers that amplify the regions of H1: 5′ ETS (external transcribed spacer); H4: 18S rRNA coding; H8: 28S rRNA coding; H13: 3′ ETS; H18, H27, H42: IGS (intergenic spacer); Prom: promoter. The *y-*axis depicts the ChIP/input ratio. Data are presented as mean ± SD (*n* = 3 independent experiments). **b** Immunoblotting shows the protein level of RPA194 remains relatively stable in Spindlin1-depleted U2OS cells ectopically expressing Spindlin1 wild type or mutants. **c** ChIP-qPCR analysis of the recruitment of RPA194 (RNA Pol I subunit) to rRNA gene promoter and coding regions (H4 and H8) in Spindlin1 KD cells, Spindlin1 KD cells expressing Myc-tagged Spindlin1 wild type or indicated mutants compared with mock cells. Results are presented as mean ± SD (*n* = 3 independent experiments). Source data are provided as a Source Data file.

We performed chromatin Immunoprecipitation quantitative real-time PCR (ChIP-qPCR) assay to analyze the association of HP1γ and Spindlin1 with various regions of rDNA repeats in Spindlin1-overexpressed HEK293T and mock cells. As shown in Fig. 4a, overexpression of Spindlin1 increased its occupancy throughout the rDNA repeats, especially at the promoter region. At the same time, a more obvious reduction of HP1γ enrichment across the rDNA loci was observed in Spindlin1-overexpressed cells comparing with that of the mock cells. Interestingly, we observed a mild reduction in the level of H3K9me3 across the rDNA repeat in Spindlin1-overexpressed cells, whereas the H3K4me3 pattern changed similarly to that of Spindlin1. These findings imply that the Spindlin1/C11orf84 complex can outcompete HP1 for the binding to H3K4me3K9me3-enriched poised rDNA loci, therefore decondense the chromatin structure for relieving transcriptional repression of rRNA genes.

### Spindlin1 binding to H3K4me3K9me3 in rDNA genes facilitates the recruitment of Pol I

We next monitored if the binding of Spindlin1/C11orf84 complex to bivalent H3K4me3K9me3 in rRNA genes could have an impact on the loading of Pol I to these bivalent chromatin domains. As shown in Fig. 4b, the protein level of Pol I subunit RPA194 did not change after Spindlin1 depletion, suggesting Spindlin1 does not affect the expression of RPA194. However, RPA194 occupancy at the rDNA promoter and coding regions was reduced in Spindlin1-depleted cells compared to control cells (Fig. 4c). Ectopic expression of Spindlin1 WT led to significantly increased occupancy of RPA194 at the rDNA promoter and coding regions whereas re-expressing F141A mutant or W62A/W72A mutant resulted in a slight increase of RPA194 occupancy. This observation further supports that binding of Spindlin1/C11orf84 complex to H3K4me3K9me3-marked rDNA promotes chromatin relaxation for elevated loading of Pol I complex, and H3K4me3K9me3-enriched rDNA repeats likely represent a poised state prior to transcriptional activation.

## Discussion

In this study, we demonstrated the functional cooperativity of Spindlin1 three Tudor domains in selective interactions with their effector proteins to regulate rRNA transcription. Spindlin1 Tudor 3 domain is responsible for the binding to C11orf84 while the other two Tudor domains act in concert to recognize a non-canonical bivalent histone mark H3K4me3K9me3. Binding to C11orf84 not only stabilizes Spindlin1 but also facilitates its association with H3K4me3K9me3. The binding of Spindlin1/C11orf84 to H3K4me3K9me2/3 likely represents among the strongest binding ever reported for multivalent readout of methylated histone marks. Functionally, the strong binding of Spindlin1/C11orf84 complex to H3K4me3K9me3 likely facilitated the displacement of HP1γ from poised chromatin, and allowed RNA Pol I machinery to gain access to these rRNA genes for transcriptional activation. As shown in ChIP-qPCR assay, overexpression of Spindlin1 reduced HP1γ occupancy at the rDNA loci compared with the control cells. Although the ChIP-qPCR assay examined a mixture of rDNA arrays including active, inactive, and poised rDNA, we reason that only those Spindlin1/C11orf84 complexes binding to H3K4me3K9me3-enriched poised rDNA loci were actually able to outcompete HP1γ for the binding of histone H3 with K4me3 and K9me3 dual marks. It is unlikely that Spindlin1/C11orf84 binds to H3K9me3-enriched constitutively silenced rDNA loci, given that Spindlin1/C11orf84 displayed an even weaker binding to H3K9me3 than HP1 proteins. On the other hand, HP1γ is likely absent at H3K4me3-marked active rDNA loci where the Spindlin1/C11orf84 complex is also localized. Our conclusion is further supported by the observation that overexpression of wild-type Spindlin1, but not its H3K4me3 or H3K9me3-binding deficient mutants, increased the Pol I occupancy on rDNA repeats and promoted rRNA transcription. Nonetheless, the high-resolution map showing the distribution of bivalent H3K4me3 and H3K9me3 mark at rDNA locus in human cells has not been reported yet. Future efforts are required to provide in vivo evidence to support our model.

H3K4me3 and H3K9me3 bivalent domains have previously been identified in several types of lineage-committed stem cells for keeping developmental genes poised for subsequent activation^6–9^. More recently, the loss and gain of bivalent domains including H3K4me3/H3K9me3 have been shown to be critical in regulating pro-metastatic drivers in melanoma^29^. These observations have raised an interesting question if Spindlin1/C11orf84 complex is actively involved in regulating these master genes through the direct recognition of bivalent H3K4me3K9me3 mark.

Hyperactive ribosome biogenesis is required to satisfy fast growth and proliferation of cancer cells^30^. Given that Spindlin1/C11orf84 complex promotes transcription of rRNA genes, it is not surprising that Spindlin1 is overexpressed in many human cancers and involved in tumor cell growth, migration, and invasion^31,32^, and knockdown of Spindlin1 suppressed cell growth and proliferation in several tumor cell lines^33,34^. Thus, our study suggests a new therapeutic strategy of blocking Spindlin1 binding to methylated H3 to inhibit tumor growth and metastasis.

## Methods

### Plasmid construction, protein expression, and purification

To generate Spindlin1/C11orf84 co-expression plasmids, DNA sequence encoding human Spindlin1 (aa: 50–262 aa) was cloned into MCS1 of pETduet-1 vector first, full-length C11orf84 and the fragment with aa: 219–305 were cloned into MCS2, respectively. Fusion proteins were expressed in *Escherichia coli* strain Rosetta DE3 (Novagen) induced by IPTG at 16 °C, and then purified by affinity chromatography, followed by size-exclusion and anion-exchange chromatography.

To generate mammalian expression plasmids for cell-based studies, human Spindlin1 or C11orf84 was cloned into a modified pCDNA3 with an N-terminal Flag tag. Spindlin2A, Spindlin2B, Spindlin3, and Spindlin4 were cloned into pCDNA3.1 vector with an N-terminal Myc tag. Spindlin1 was also cloned into the pEGFP-C1 vector (Clontech) for fluorescence microscopy. Mutagenesis to introduce point or truncated mutation was performed using QuikChange II XL site-directed mutagenesis kit (Agilent Genomics). The mutagenesis was also carried out to generate the shRNA resistant plasmids in rescue assays. The original sequence targeted by the shRNA and the mutated sequence for shRNA resistance in rescue experiments are listed in Supplementary Table 4.

### Limited proteolysis-coupled mass spectrometry

The purified complex of Spindlin1 (aa: 50–262) with full-length C11orf84 was digested with individual proteolytic enzyme including Elastase (1:1000, w/w), Trypsin (1:2000, w/w), and proteinase K (1:10,000, w/w) at 25 °C for 1 h. The proteolytic product was separated by size-exclusion chromatography and visualized by sodium dodecyl sulfate-polyacrylamide gel electrophoresis (SDS-PAGE) gel; SDS-PAGE gel bands of the factions from major gel filtration peaks were further analyzed by liquid chromatography–mass spectrometry.

### Isothermal titration calorimetry

Calorimetric experiments were conducted at 25 °C with a MicroCal ITC200 microcalorimeter (GE Healthcare) following the standard procedure. The ITC buffer contains 20 mM HEPES pH 7.5 and 150 mM NaCl. Titrations were performed as follows: 50 μM Spindlin1 or Spindlin1/C11orf84 complex protein solution was transferred into the sample cell, one preliminary injection of 0.5 μl of 0.8 mM H3K4me3K9me3 peptide was followed by about 30 injections of 1 μl. A 2-min delay between the injections was applied to allow the system to reach equilibrium. Data were analyzed using MicroCal Origin 7.0 software. Sequences of H3 peptides used in ITC-binding assays are shown in Supplementary Table 5.

### Crystallization and structure determination

Crystals of Spindlin1/C11orf84/H3K4me3K9me3 ternary complex were obtained at 291 K with the vapor diffusion hanging drop method by mixing 1:1.5 molar ratio Spindlin1 (aa: 50–262)/C11orf84 (aa: 253–295) binary complex with H3K4me3K9me3 (A1-G12) peptide in the crystallization buffer (0.1 M sodium cacodylate pH 6.5, 35% PEG3350, 5% glycerol, 2% benzamidine hydrochloride). All diffraction data were collected at 100 K at SSRF beamline BL17U with a wavelength of 0.97907 Å. All data were processed with XDS^35^. The complex structure was solved by molecular replacement using a previously determined Spindlin1 structural model (PDB code: 4MZF) as the template. The molecular replacement solution was used subsequently for the structure refinement using Phenix and COOT^36,37^. All structure figures were generated using Pymol (Schrödinger, LLC; http://www.pymol.org). X-ray data collection and refinement statistics are listed in Supplementary Table 2.

### Cell culture and stable cell line establishment

HEK293T and U2OS cells were cultured in Dulbecco’s modified Eagle’s medium (DMEM) and McCoy’s 5A media, respectively, supplemented with 10% FBS at 37 °C with 5% CO_2_. To establish Spindlin1 or C11orf84 stably overexpressed HEK293T and U2OS cell lines, the transfected cells were selected with 1 μg/ml puromycin. To establish Spindlin1 or C11orf84 stable knockdown cell line, U2OS cells were transfected by pGPU6 vector-based shRNA plasmids purchased from GenePharma (Shanghai) followed by selection in medium with 500 μg/ml G418.

### Antibodies

The following antibodies were used in this study: anti-Flag M2 mouse monoclonal antibody (Sigma, F1804); anti-Flag rabbit monoclonal antibody (Proteintech, 20543-1-AP); anti-Myc mouse monoclonal antibody (Sigma, 9E10); anti-β-actin mouse monoclonal antibody (Cell Signaling Technology, 8H10D10); anti-RPA194 C-1 mouse monoclonal antibody (Santa Cruz, sc-48385); anti-nucleolin antibody produced in rabbit (Sigma, N2662); anti-SPIN1 rabbit polyclonal antibody (Proteintech, 12105-1 AP); anti-C11orf84 rabbit polyclonal antibody (Sigma, HPA040128); anti-Histone H3 (tri-methyl K4) rabbit antibody (Abcam, ab8580); anti-Histone H3 (tri-methyl K9) rabbit antibody (Abcam, ab8898); anti-HP1gamma, clone 42s2 antibody (Millipore, 05-690).

### Coimmunoprecipitation

Flag- and Myc-tagged Spindlin expression plasmids were co-transfected into HEK293T cells using polyethylenimine. The cells were lysed in NP40 buffer (50 mM Tris pH 7.5, 150 mM NaCl, 1 mM EDTA, and 1% NP40) supplemented with PMSF and protease inhibitor cocktail (Roche). Clarified cell lysates were incubated with anti-Flag M2 beads (Sigma) for 1 h. Immunoprecipitated proteins were eluted after washing beads three times.

### Histone peptide pull down assay

Flag-Spindlin1 and Myc-C11orf84 were transiently transfected alone or co-transfected into HEK293T cells. The cells were lysed in NP40 supplemented with PMSF and protease inhibitor cocktail (Roche). Strep-tactin beads (GE Healthcare) were washed three times with NP40 buffer before incubated with 100 μg biotin-labeled H3K4me3R8me2a peptide (The peptide sequence as detailed in Supplementary Table 5.) After incubation for 1 h at 4 °C in a rocking rack, the beads were washed three times with NP40 buffer and then eluted with 100 μl 2× SDS loading buffer. Five percent cell lysates were reserved as the input. Both the input and eluted samples were run on 10% SDS-PAGE gel and probed by immunoblotting.

### Immunoblotting

Proteins from total cell lysate or protein samples from in vitro pull-down or co-IP denatured in protein loading buffer (Bio-Rad) were resolved on 10% SDS-PAGE gel and then transferred to PVDF membrane (Millipore). The membrane was blocked by 5% non-fat milk in TBS-tween 20 (TBST) buffer for 1 h at room temperature. The membrane was then incubated with primary antibodies at 4 °C overnight with mild shaking. After washing three times with TBST buffer, the membrane was incubated with HRP-conjugated goat anti-mouse or anti-rabbit secondary antibody (GE Healthcare) for 1 h at room temperature. The protein bands were then detected by Western Lightning Plus-ECL (Perkin Elmer) after washing with TBST three times. The membrane was imaged on the ChemiDoc MP system (Bio-Rad).

### Immunofluorescence microscopy

U2OS cells were inoculated on glass coverslips and transfected with indicated plasmids for 48 h. The transfected cells were fixed with 4% paraformaldehyde in PBS for 5 min and permeabilized with 0.2% Triton X-100, 0.04% SDS in PBS for 5 min. After blocking in 10% normal goat serum (Invitrogen) at room temperature for 30 min, cells were incubated overnight at 4 °C with indicated primary antibodies. The appropriate Alexa-Fluor 488 or Cy3-coupled secondary antibody (Invitrogen) was applied for 1 h at room temperature. The cells were mounted in ProLong-Gold antifade Mountant with 6-diamidino-2-phenylindole (Life Technologies) and imaged with a ZSM 780 laser confocal microscope (Carl Zeiss).

### ChIP-qPCR

ChIP was performed with EZ ChIP Kit (Millipore) according to the manufacturer’s protocol. Briefly, HEK293T or U2OS cells were crosslinked with 1% formaldehyde for 10 min at room temperature and quenched crosslinking with 0.125 M glycine for 5 min. Nuclei was isolated using cell lysis buffer (20 mM Tris-HCl pH 8.0, 85 mM KCl, 0.5% NP40). The nuclear fraction was then digested using MNase (New England Biolabs). Digestion was stopped with 0.05 M EDTA. The sheared chromatin samples were incubated overnight at 4 °C with antibodies against Spindlin1, HP1γ, H3K4me3, and H3K9me3, respectively. Ten percent of the samples were reserved as the input control. Protein A/G magnetic beads were then incubated with the chromatin-antibody mixtures on a rotating rack for 2 h at 4 °C. The beads were washed sequentially with washing buffers provided by the kit according to the manufacturer’s manual. Crosslinking was reversed by heating for 16 h at 65 °C followed by proteinase K treatment at 55 °C for 1 h and RNase A treatment at 37 °C for 30 min, respectively. The DNA fragments were then purified with spin columns provided with the kit. The purified DNA fragments were quantified by qPCR reactions using ChamQTM SYBR qPCR Master Mix (Vazyme) according to the supplier’s protocol. All the ChIP-qPCR primers are listed in Supplementary Table 6.

### RNA extraction, RT-PCR, and quantitative PCR (qPCR)

Total RNA was extracted from cells using RNAiso plus reagent (Takara). Reverse transcription was conducted using the PrimeScript® RT reagent Kit (Takara) following the manufacturer’s manual. qPCR reactions were carried out with StepOnePlus real-time PCR System (Applied Biosystems) using ChamQTM SYBR® qPCR Master Mix (Vazyme). The relative expression level of 45S pre-rRNA as well as each SPIN family gene was calculated with 2^(−ΔΔCt)^ method. The two-tailed *t*-tests were performed to calculate corresponding *p* values. All the RT-qPCR primers are listed in Supplementary Table 6.

### Cell proliferation assay

Cell counting kit-8 (CCK-8) assay (Dojindo) was used to measure cell proliferation following the manufacturer’s guidelines. Briefly, cells were seeded in 96-well plates (1000 cells per well) and cultured for the indicated days. To measure cell number, 10 µl CCK-8 was added into each well, followed by continuous incubation for 4 h. The optical density value of cells in each well was measured at the wavelength of 450 nm on a microplate reader. The assay was carried out in triplicates and repeated three times.

### Reporting summary

Further information on research design is available in the Nature Research Reporting Summary linked to this article.

## Supplementary information

Supplementary Information

Reporting Summary

## Data Availability

The coordinate and structure factor for the reported crystal structure has been deposited in the Protein Data Bank with the following accession code: 7CNA. All data are available from the corresponding author upon reasonable request. Source data are provided with this paper.

## Source data

Source Data
