# Supplementary material for: Structural mechanism of bivalent histone H3K4me3K9me3 recognition by the Spindlin1/C11orf84 complex in rRNA transcription activation
Source: Nat Commun. 2021 Feb 11;12:949. doi: 10.1038/s41467-021-21236-x (PMC7878818; doi:10.1038/s41467-021-21236-x)
Supplement: Supplementary file 1 — Supplementary Information [file 41467_2021_21236_MOESM1_ESM.pdf]

Supplementary Information

Structural Mechanism of Bivalent Histone H3K4me3K9me3 Recognition by the Spindlin1/C11orf84 Complex in rRNA Transcription Activation

Yongming Du[1], Yinxia Yan[1], Si Xie[1], Hao Huang[2], Xin Wang[2], Ray Kit Ng[1], Ming-Ming Zhou[3], Chengmin Qian[1]*

[1]School of Biomedical Sciences, The University of Hong Kong, Hong Kong

[2]Department of Biomedical Sciences, The City University of Hong Kong, Hong Kong

[3]Department of Pharmacological Sciences, Icahn School of Medicine at Mount Sinai, New York, NY, USA.

* Correspondence: cmqian@hku.hk

This Supplementary Information file contains:

- **Supplementary Figure 1-5**
- **Supplementary Table 1-6**

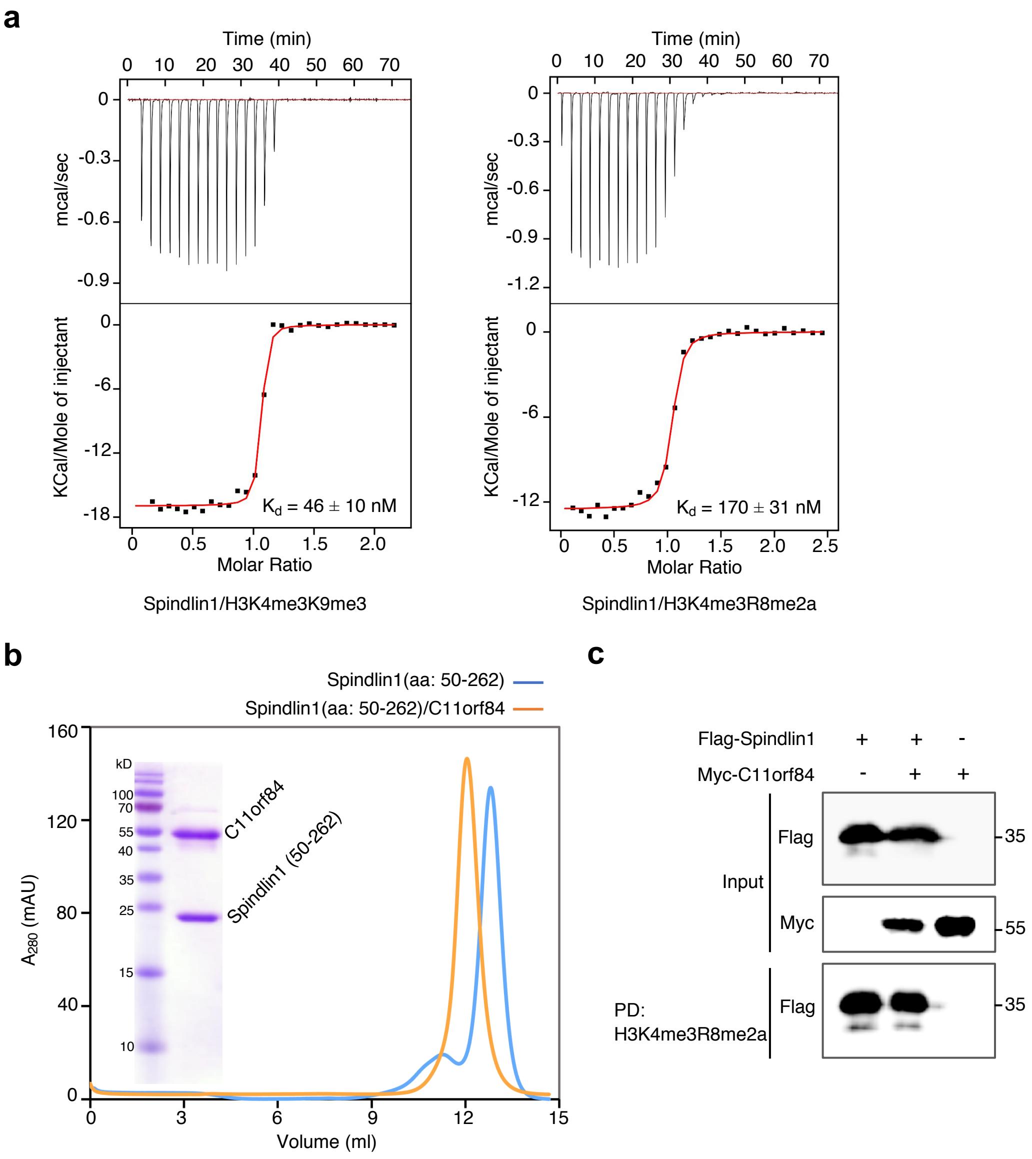

Supplementary Figure 1. Non-canonical bivalent H3K4me3K9me3 binding by Spindlin1 in complex with C11orf84. (a) The representative ITC curves of titrating H3K4me3K9me3 and H3K4me3R8me2a peptides to Spindlin1 protein solution respectively. (**b**) Size exclusion chromatography of Spindlin1 (aa: 50-262) alone and in complex with full-length C11orf84. The SDS-PAGE gel of the peak fraction from the Spindlin1/C11orf84 complex suggested Spindlin1 and C11orf84 form a heterodimer. (**c**) *In vitro* pull-down assay showed that overexpressing Myc-tagged C11orf84 does not disrupt the binding of Flag-tagged Spindlin1 to biotinylated H3K4me3R8me2a peptide. PD: pull down. The immunoblotting was repeated twice with similar results.

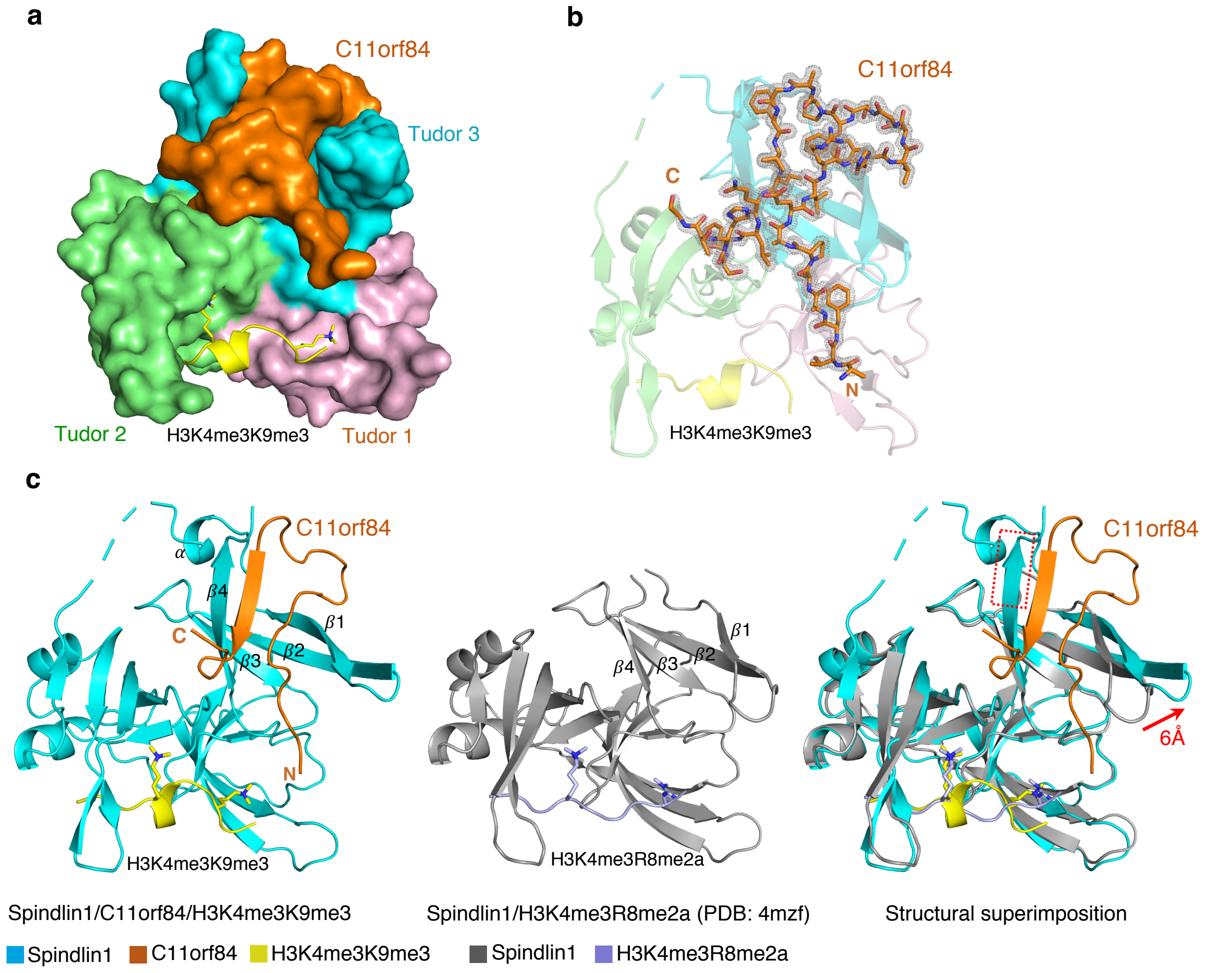

Supplementary Fig 2. Structural Comparison of Spindlin1/C11orf84/H3K4me3K9me3 ternary complex with the previously determined Spindlin1/H3K4me3R8me2a binary complex (PDB: 4MZF). (**a**) Surface representation of Spindlin1/C11orf84/H3K4me3K9me3 ternary complex. Spindlin1 Tudor 1, Tudor 2 and Tudor 3 domains are coloured in light pink, green and cyan, respectively. The C11orf84 segment and H3K4me3K9me3 peptide are coloured in orange and yellow respectively. (**b**) Electron density map of the C11orf84 segment in the ternary complex. 2Fo-Fc map is contoured at the 1.5σ level and coloured in light grey. **(c)** Structural superimposition gives a RMSD of 0.7 Å for all backbone atoms. Binding of C11orf84 stabilized Spindlin1 structure as the region A204-D211 including a N-terminal α helix of Tudor 3 domain is clearly defined in our structure but invisible in the previous determined Spindlin1 structures. The binding also caused major conformational changes in Spindlin1 Tudor 3 domain: The red dash box highlights the Y256-K260 segment of Tudor 3 adopts a β strand conformation upon C11orf84 binding. The β1-loop-β2 region is flipped outward with ~6 Å in distance to accommodate the C11orf84 fragment, as indicated by the red

arrow. Spindlin1, C11orf84 and H3K4me3K9me3 in the ternary complex are coloured in cyan, orange and yellow respectively. Spindlin1 and H3K4me3R8me2a in Spindlin1/H3K4me3R8me2a binary complex are coloured in light grey and blue respectively.

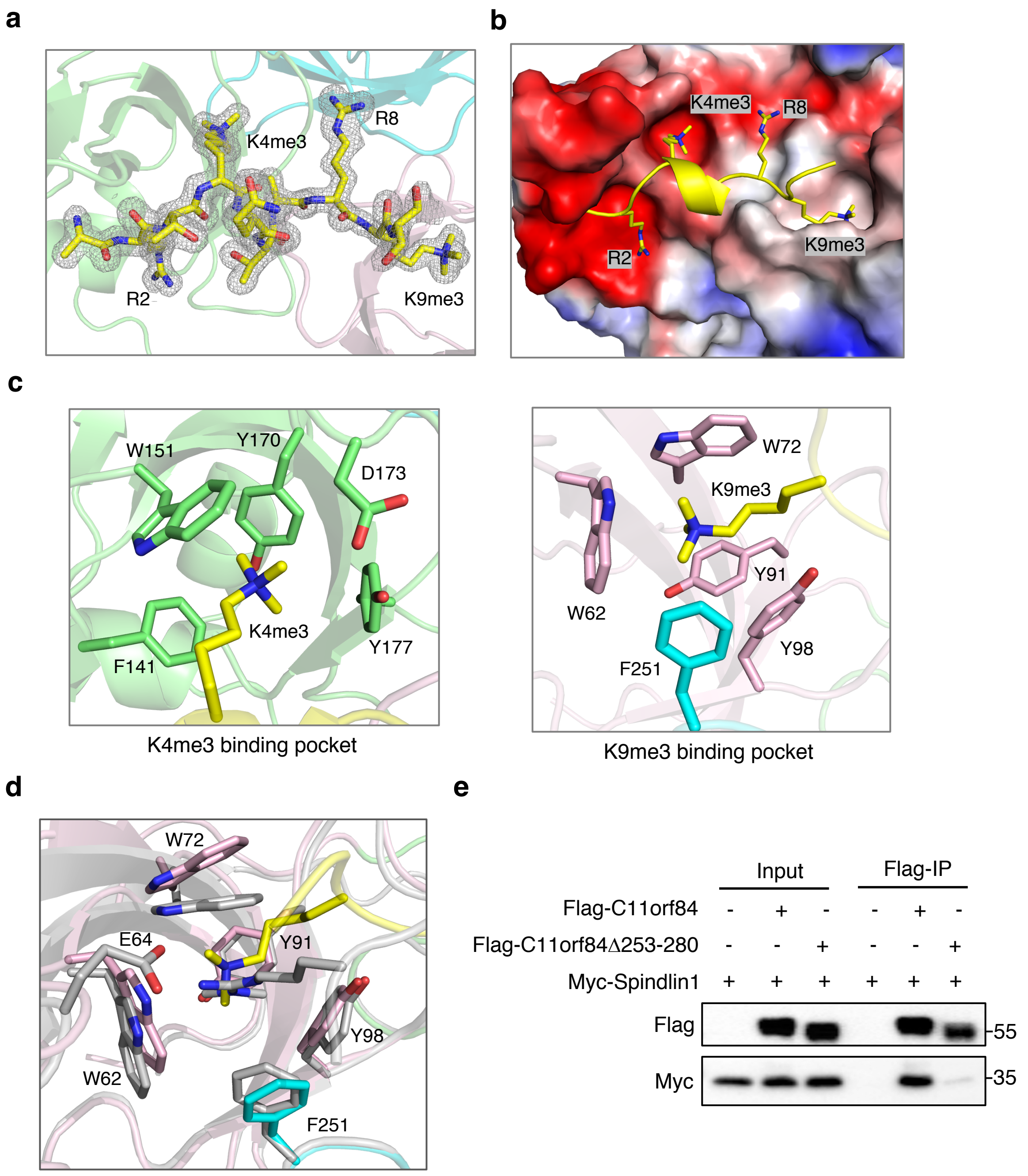

Supplementary Figure 3. Detailed interactions between H3K4me3K9me3 and Spindlin1/C11orf84 complex. (**a**) Electron density map of the H3K4me3K9me3 peptide in the ternary complex. 2Fo-Fc map contoured at the 1.5σ level is shown as light grey mesh. (**b**) Electrostatic potential surface of H3K4meK9me3 binding site on Spindlin1. The critical histone H3 residues are depicted in stick model. (**c**) Positioning of K4me3 and K9me3 in the aromatic pockets of Tudor2 and Tudor1 respectively. K4me3 in the ternary complex is inserted into a pocket formed by residues F141, W151, Y170 and Y177, which is the same as the previously determined Spindlin1/H3K4me3R8me2a binary complex (PDB: 4MZF). K9me3 in

our present Spindlin1/C11orf84/H3K4me3K9me3 complex is positioned in a pocket formed by residues W62, W72, Y91, Y98 and F251, of which Y91 forms the base and W62, W72, Y98 and F251 form the wall. (**d**) Comparison of K9me3 binding pocket in our ternary complex with the one to accommodate R8me2a in Spindlin1/H3K4me3R8me2a (PDB: 4MZF, coloured in grey) indicates these aromatic residues have minor conformational rearrangements. Intriguingly, residue E64 that participated in R8me2a recognition is not involved in K9me3 binding in our complex structure. (**e**) Co-IP assay showed that C11orf84Δ253-280 truncation impaired its interaction with Spindlin1, therefore confirmed our structural finding that amino acid sequence 253-280 of C11orf84 is required for Spindlin1 binding.

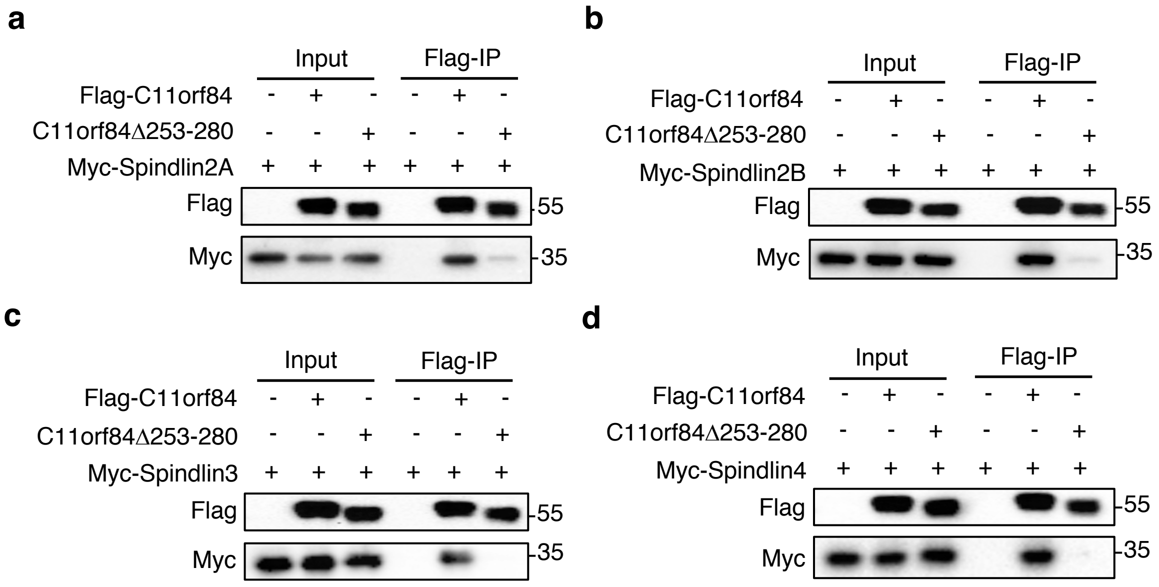

e

```
Spindlin1    MKTPFGKT-PGQRSRADAGHAGVSANMMKKRTSHKKHRSSVGPSKPVSQPRRNIVGCRIQ 59
Spindlin2A   MKTPNAQEAEGQQTRAAAGRATGSANMTKKKVSQKKQRG-----RPSSQPCRNIVGCRIS 55
Spindlin2B   MKTPNAQEAEGQQTRAAAGRATGSANMTKKKVSQKKQRG-----RPSSQPRRNIVGCRIS 55
Spindlin3    MKTPFGKAAAGQRSRTGAGHGSVSVTMIKRKAAHKKHRS-----RPTSQPRGNIVGCRIQ 55
Spindlin4    ---------MSPPTVPPMGVDGVSAYLMKKRHTHRKQRR-----KPTFLTRRNIVGCRIQ 46
                              .  :    *    *. : *:: :::*:*      :*      *******.
                                                NoLS R/K motif

Spindlin1    HGWKEGNGPVTQWKGTVLDQVPVNPSLYLIKYDGFDCVYGLELNKDERVSALEVLPDRVA 119
Spindlin2A   HGWKEGDEPITQWKGTVLDQVPINPSLYLVKYDGIDCVYGLELHRDERVLSLKILSDRVA 115
Spindlin2B   HGWKEGDEPITQWKGTVLDQVPINPSLYLVKYDGIDCVYGLELHRDERVLSLKILSDRVA 115
Spindlin3    HGWKDGDEPLTQWKGTVLDQVPVNPSLYLIKYDGFDCVYGLELHRDERVSSLEVLPNRVA 115
Spindlin4    HGWKEGNEPVEQWKGTVLEQVSVKPTLYIIKYDGKDSVYGLELHRDKRVLALEILPERVP 106
             ****:*:  *:  *******:** ::*:**::**** *.******::*:** :*::* :**
                               H3K9me3 pocket

Spindlin1    TSRISDAHLADTMIGKAVEHMFETEDGSKDEWRGMVLARAPVMNTWFYITYEKDPVLYMY 179
Spindlin2A   SSHISDANLANTIIGKAVEHMFEGEHGSKDEWRGMVLAQAPIMKAWFYITYEKDPVLYMY 175
Spindlin2B   SSHISDANLANTIIGKAVEHMFEGEHGSKDEWRGMVLAQAPIMKAWFYITYEKDPVLYMY 175
Spindlin3    SSRISDTHLAEIMVGKAVEHIFETEEGSKNEWRGMVLAQAPVMNTWFYITYEKDPVLYMY 175
Spindlin4    TPRI-DSRLADSLIGKAVEHVFEGEHGTKDEWKGMVLARAPVMDTWFYITYEKDPVLYMY 165
             : :* *:.**: ::******:** *.*:*:**:*****:**:*.:***************
                                          H3K4me3 pocket

Spindlin1    QLLDDYKEGDLRIMPDSND-SPPAEREPGEVVDSLVGKQVEYAKEDGSKRTGMVIHQVEA 238
Spindlin2A   QLLDDYKEGDLRIMPESSE-SPPTEREPGGVVDGLIGKHVEYTKEDGSKRIGMVIHQVET 234
Spindlin2B   QLLDDYKEGDLRIMPESSE-SPPTEREPGGVVDGLIGKHVEYTKEDGSKRIGMVIHQVEA 234
Spindlin3    QLLDDYKDGDLRILQDSND-SPLAEREPGEVIDSLVGKQVEYAKDDGSKRTGMVIHQVEA 234
Spindlin4    TLLDDYKDGDLRIIPDSNYYFPTAEQEPGEVVDSLVGKQVEHAKDDGSKRTGIFIHQVVA 225
              ******:*****: :*.   * :*:*** *:*.*:**:**::*:***** *:.**** :
                                                          Tudor 3

Spindlin1    KPSVYFIKFDDDFHIYVYDLVKTS  262
Spindlin2A   KPSVYFIKFDDDFHIYVYDLVKKS  258
Spindlin2B   KPSVYFIKFDDDFHIYVYDLVKKS  258
Spindlin3    KPSVYFIKFDDDFHIYVYDLVKTS  258
Spindlin4    KPSVYFIKFDDDIHIYVYGLVKTP  249
             ************:*****.***.
```

Supplementary Figure 4. C11orf84 interacts with Spindlin family proteins. HEK293T cells were co-transfected with Flag-tagged C11orf84 and (**a**) Myc-tagged Spindlin2A, (**b**) Myc-

tagged Spindlin2B, (**c**) Myc-tagged Spindlin3, (**d**) Myc-tagged Spindlin4. Cells were lysed and subjected to Flag-IP. The input and IP eluted samples were examined by immunoblotting probed with indicated antibodies. (**a**-**d**) The immunoblotting presents a representative result from two independent experiments. (**e**) Sequence alignment of all human Spindlin family proteins. * indicates positions with fully conserved residues; : indicates conservation between groups of strongly similar properties; . indicates conservation between groups of weakly similar properties. The R/K rich NoLS sequences of Spindlin proteins are indicated. Residues forming H3K4me3 and H3K9me3 binding pockets are highlighted in bold and colored in blue and purple, respectively.

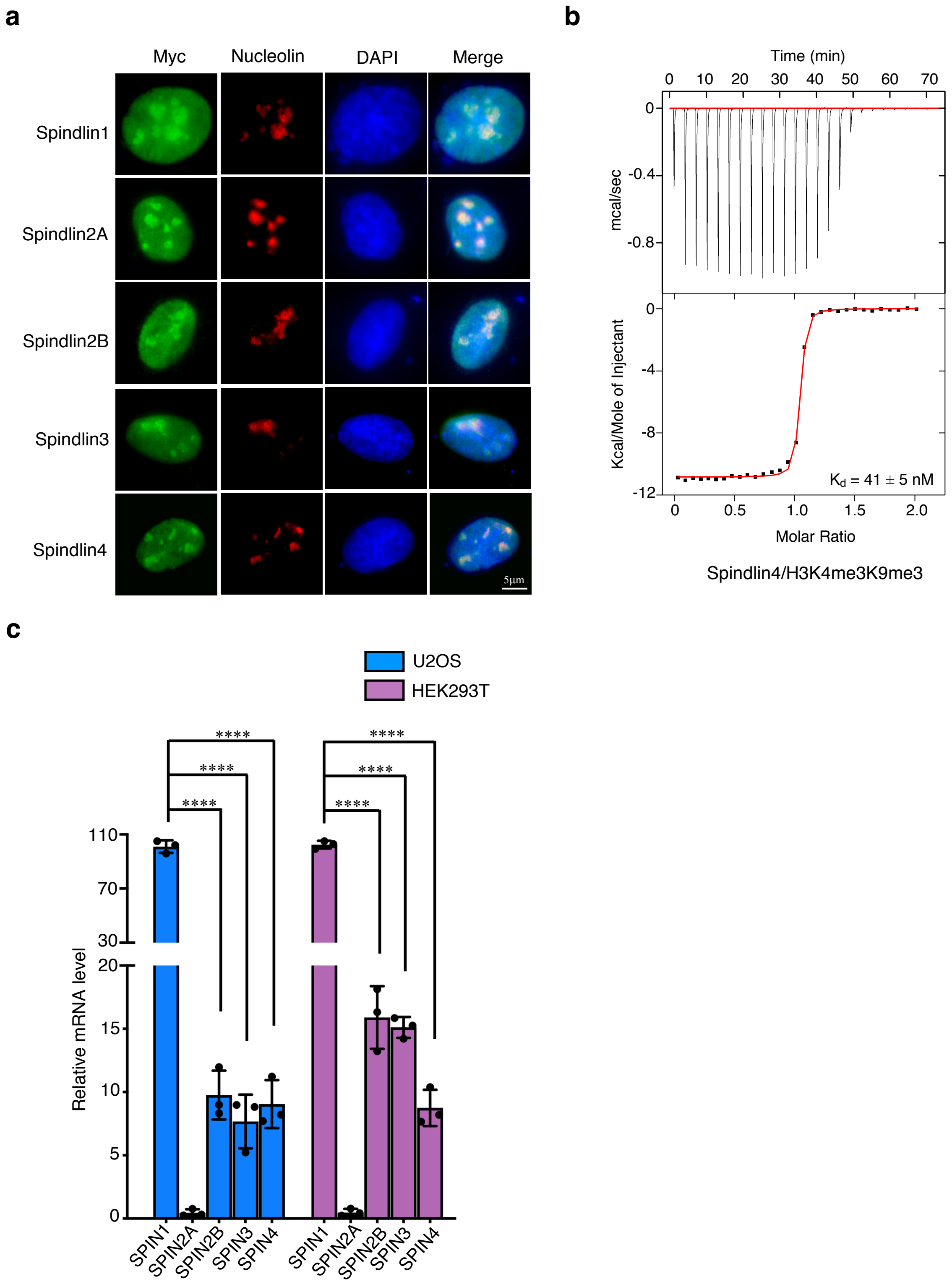

Supplementary Figure 5. Cellular localization and the expression level of Spindlin family members. (**a**) Cellular localizations of Spindlin family members indicated by immunofluorescence microscopy. Myc-tagged Spindlin paralogs expressed in U2OS cells were subject to immunostaining. Nucleolin (NCL) was immunostained as a nucleolar marker. Nuclear DNA was stained with DAPI. Scale bar, 5 μm. (**b**) ITC measurement shows Spindlin4

binds to H3K4me3K9me3 with a K_d of 41 ± 5 nM, similar to that of Spindlin1. (**c**) Quantification of human *SPIN* gene mRNA levels in U2OS cells and HEK293T cells. Total cDNA from U2OS cells or HEK293T cells was applied to qPCR to measure mRNA levels of *SPIN1*, *SPIN2A*, *SPIN2B*, *SPIN3* and *SPIN4*. The GAPDH gene is used as internal control to normalize the mRNA levels of *SPIN* gene members. Relative mRNA levels of *SPIN2A*, *SPIN2B*, *SPIN3* and *SPIN4* are presented as the percentage of *SPIN1*. Values are mean ± SD for three independent biological repeats. **** $p < 0.0001$ (unpaired t-test, two tailed).

Supplementary Table 1. Summary of the enthalpy changes (ΔH ± SD), entropy changes (TΔS), binding affinities (K_d ± SD) and binding stoichiometry (N) of the ITC measurements of various H3 peptides binding to Spindlin1 and Spindlin1/C11orf84 complex.

Protein	Peptide: H3 (1-12)	N	ΔH (kcal/mol)	TΔS (kcal/mol)	Kd (nM)
Spindlin1	H3K4me3R8me2a	1.02	-11.87 ± 0.09	-3.22	170 ± 31
	H3K4me3K9me3	1.04	-17.18 ± 0.13	-7.13	46 ± 10
Spindlin1/C11orf84	H3K4me3	1.01	-10.85 ± 0.08	-1.72	213 ± 33
	H3K9me3	1.02	-10.02 ± 0.21	-2.47	2880 ± 401
	H3K4me3R8me2a	1.04	-12.50 ± 0.03	-2.84	82 ± 12
	H3K4me3K9me2	1.01	-15.67 ± 0.13	-5.58	42 ± 13
	H3K4me3K9me3	1.02	-15.76 ± 0.14	-5.19	20 ± 11

Supplementary Table 2. X-ray crystallographic data collection and refinement statistics.

	Spindlin1/c11orf84/H3K4me3K9me3
**Data collection**	
Beam line	SSRF-BL17U
Wavelength (Å)	0.97907
Space group	$P12_11$
Cell dimensions	
a, b, c (Å)	45.60, 101.18, 56.62
α, β, γ (°)	90.00, 91.41, 90.00
Resolution (Å)	50.0-1.60 (1.66-1.60)*
R_{sym} or R_{merge}	0.026 (0.11)
I / σI	32.7 (10.7)
Completeness (%)	98.7 (98.8)
Redundancy	3.2 (3.2)
**Refinement**	
Resolution (Å)	28.30-1.60
No. reflections	66720 (6664)
R_{work} / R_{free}	18.3 / 20.8
No. atoms	4230
Protein	3890
Ligand/ion	57
Water	283
B-factors	22.2
Protein	21.9
Ligand/ion	26.1
Water	25.2
R.m.s. deviations	
Bond lengths (Å)	0.007
Bond angles (°)	1.10
Ramachandran statistics (Favoured/ allowed/ outliers)	98.3/1.7/0

*Values in parentheses are for highest-resolution shell.

Supplementary Table 3. Isothermal titration calorimetry parameters of titrating the H3K4me3K9me3 peptide to Spindlin1(mutants)/C11orf84 complex.

Spindlin1 mutant	N	ΔH (kcal/mol)	TΔS (kcal/mol)	K_d (nM)
W62A	1.06	-14.29 ± 0.11	-4.50	71 ± 14
W72A	1.07	-14.05 ± 0.13	-4.30	77 ± 18
Y91A	1.07	-14.88 ± 0.11	-4.98	59 ± 11
Y98A	1.07	-14.70 ± 0.11	-5.01	80 ± 16
F141A	1.03	-7.19 ± 0.08	-1.09	869 ± 110
D173A	1.04	-14.36 ± 0.10	-4.41	57 ± 11
W62A/W72A	1.00	-10.58 ± 0.04	-1.43	199 ± 15

Supplementary Table 4. Sequence of shRNA (targeting) used in this study.

si/shRNA name	Sequences (5'-3')
shSPIN1	Targeting sequence: GAATATGCCAAAGAAGAT<br>shRNA resistant: GAGTACGCTAAGGAGGAC
shC11orf84	Targeting sequence: GCTTCGTCTTGCAGCTCTTC<br>shRNA resistant: GATTTGTATTTACAACTATTT

Supplementary Table 5. Sequence of histone H3 peptides used in this study.

Peptide name	Sequences (N-C)	Application
H3K4me3	ARTKme3QTARKSTG	ITC
H3K9me3	ARTKQTARKme3STG	ITC
H3K4me3R8me2a	ARTKme3QTARme2aKSTG	ITC, crystallization
H3K4me3R8me2a-biotin	ARTKme3QTARme2aKSTGGK-biotin	Pull down assay
H3K4me3K9me2	ARTKme3QTARKme2STG	ITC
H3K4me3K9me3	ARTKme3QTARKme3STG	ITC, crystallization

Supplementary Table 6. Sequence of qPCR primers used in this study.

**Primer name**	**Sequences (5'-3')**	**Application**
GAPDH	Sense: GGAGCGAGATCCCTCCAAAAT<br>Antisense: GGCTGTTGTCATACTTCTCATGG	RT-qPCR
45S pre-rRNA	Sense: GCCTTCTCTAGCGATCTGAGAG<br>Antisense: CCATAACGGAGGCAGAGACA	
SPIN1	Sense: ATGAAGACCCCATTCGGAAA<br>Antisense: TGTGGGATGTCCTCTTCTTC	
SPIN2A	Sense: TTGCAAGCAGTCACTCAGCG<br>Antisense: CTTCATGCCTGCTCGATGCT	
SPIN2B	Sense: CATGAAGACCCCCAACGCA<br>Antisense: GTCGGCCTCTCTGCTTCTTT	
SPIN3	Sense: ACTGCACCGCGATAAGAGAG<br>Antisense: CAAACACATGCTCCACTGCC	
SPIN4	Sense: GTCCGATTTGTGCACTTGAGG<br>Antisense: GCCTTTCCAAACGGGGTCTT	
Promoter	Sense: GAGGTATATCTTTCGCTCCGAGTC<br>Antisense: CAGCAATAACCCGGCGG	ChIP-qPCR
H1	Sense: GGCGGTTTGAGTGAGACGAGA<br>Antisense: ACGTGCGCTCACCGAGAGCAG	
H4	Sense: CGACGACCCATTCGAACGTCT<br>Antisense: CTCTCCGGAATCGAACCCTGA	
H8	Sense: AGTCGGGTTGCTTGGGAATGC<br>Antisense: CCCTTACGGTACTTGTTGACT	
H13	Sense: ACCTGGCGCTAAACCATTCGT<br>Antisense: GGACGGGCCCTTGTGTCGAGG	
H18	Sense: GTTGACGTACAGGGTGGACTG<br>Antisense: GGAAGTTGTCTTCACGCCTGA	
H27	Sense: CCTTCCACGAGAGTGAGAAGCG<br>Antisense: CTCGACCTCCCGAAATCGTACA	
H42	Sense: GGTTGTCGGGCTCCATCT<br>Antisense: CTTTCCGGAGCTCTGCCTAG	
