# Supplementary material for: Structural mechanism of bivalent histone H3K4me3K9me3 recognition by the Spindlin1/C11orf84 complex in rRNA transcription activation
Source: Nat Commun. 2021 Feb 11;12:949. doi: 10.1038/s41467-021-21236-x (PMC7878818; doi:10.1038/s41467-021-21236-x)
Supplement: Supplementary file 2 — Reporting Summary [file 41467_2021_21236_MOESM2_ESM.pdf]

nature research

Corresponding author(s): Chengmin Qian
Last updated by author(s): 2021-1-3

Reporting Summary

Nature Research wishes to improve the reproducibility of the work that we publish. This form provides structure for consistency and transparency in reporting. For further information on Nature Research policies, see our Editorial Policies and the Editorial Policy Checklist.

Statistics

For all statistical analyses, confirm that the following items are present in the figure legend, table legend, main text, or Methods section.

n/a	Confirmed	
☐	☒	The exact sample size (*n*) for each experimental group/condition, given as a discrete number and unit of measurement
☐	☒	A statement on whether measurements were taken from distinct samples or whether the same sample was measured repeatedly
☐	☒	The statistical test(s) used AND whether they are one- or two-sided<br>*Only common tests should be described solely by name; describe more complex techniques in the Methods section.*
☒	☐	A description of all covariates tested
☒	☐	A description of any assumptions or corrections, such as tests of normality and adjustment for multiple comparisons
☐	☒	A full description of the statistical parameters including central tendency (e.g. means) or other basic estimates (e.g. regression coefficient) AND variation (e.g. standard deviation) or associated estimates of uncertainty (e.g. confidence intervals)
☐	☒	For null hypothesis testing, the test statistic (e.g. *F*, *t*, *r*) with confidence intervals, effect sizes, degrees of freedom and *P* value noted<br>*Give P values as exact values whenever suitable.*
☒	☐	For Bayesian analysis, information on the choice of priors and Markov chain Monte Carlo settings
☒	☐	For hierarchical and complex designs, identification of the appropriate level for tests and full reporting of outcomes
☒	☐	Estimates of effect sizes (e.g. Cohen's *d*, Pearson's *r*), indicating how they were calculated

Our web collection on statistics for biologists contains articles on many of the points above.

Software and code

Policy information about availability of computer code

Data collection	The diffraction data of this study was collected from Shanghai Synchrotron Radiation Facility (SSRF) beamline BL17U.
Data analysis	1. Diffraction data was indexed, integrated and scaled with XDS Version January 26, 2018 (Kabsch W., 2010).<br>2. The crystal structure was solved with Phenix 1.12 (Adams et al., 2010) and Coot 0.8.6 (Emsley et al., 2010).<br>3. All structure figures were created using PyMOL V2.0 (DeLano Scientific LLC (Schrödinger, LLC)).<br>4. All the Isothermal titration calorimetry data were analyzed using MicroCal Origin 7.0.<br>5. The data from qPCR and CCK-8 assay were analyzed using Prism Graphpad 8.4.0.

For manuscripts utilizing custom algorithms or software that are central to the research but not yet described in published literature, software must be made available to editors and reviewers. We strongly encourage code deposition in a community repository (e.g. GitHub). See the Nature Research guidelines for submitting code & software for further information.

Data

Policy information about availability of data

All manuscripts must include a data availability statement. This statement should provide the following information, where applicable:

- Accession codes, unique identifiers, or web links for publicly available datasets
- A list of figures that have associated raw data
- A description of any restrictions on data availability

The coordinate and structure factor for the reported crystal structure have been deposited in the Protein Data Bank with the following accession code: 7CNA. Crystallographic data collection and refinement statistics are supplied as Supplementary Table 2. A source data file containing the raw data is provided.

Field-specific reporting

Please select the one below that is the best fit for your research. If you are not sure, read the appropriate sections before making your selection.

☒ Life sciences ☐ Behavioural & social sciences ☐ Ecological, evolutionary & environmental sciences

For a reference copy of the document with all sections, see nature.com/documents/nr-reporting-summary-flat.pdf

Life sciences study design

All studies must disclose on these points even when the disclosure is negative.

Sample size	No sample size calculation was performed. Independent biological repeats followed by statistical analysis were performed to confirm the results.
Data exclusions	No data was excluded.
Replication	All quantitative PCR data presented in this manuscript were repeated three times. The immunoblotting and fluorescence microscopy in this manuscript were repeated twice with similar results. These data can be repeated successfully.
Randomization	Randomization was not used since there are no experimental groups.
Blinding	No Blinding was performed, since no subjective variance is involved in this study.

Reporting for specific materials, systems and methods

We require information from authors about some types of materials, experimental systems and methods used in many studies. Here, indicate whether each material, system or method listed is relevant to your study. If you are not sure if a list item applies to your research, read the appropriate section before selecting a response.

Materials & experimental systems

n/a	Involved in the study	
☐	☒	Antibodies
☐	☒	Eukaryotic cell lines
☒	☐	Palaeontology and archaeology
☒	☐	Animals and other organisms
☒	☐	Human research participants
☒	☐	Clinical data
☒	☐	Dual use research of concern

Methods

n/a	Involved in the study	
☒	☐	ChIP-seq
☒	☐	Flow cytometry
☒	☐	MRI-based neuroimaging

Antibodies

Antibodies used	anti-Flag M2 mouse monoclonal antibody (Sigma, F1804), 1:1000 dilution for immunoblotting.<br>anti-Flag rabbit monoclonal antibody (Proteintech, 20543-1-AP), 1:1000 dilution for immunoblotting.<br>anti-Myc mouse monoclonal antibody (Sigma, 9E10), 1:1000 dilution for immunoblotting<br>anti-β-actin mouse monoclonal antibody (Cell Signaling Technology, 8H10D10), 1:2000 dilution for immunoblotting<br>anti-RPA194 C-1 mouse monoclonal antibody (Santa Cruz, sc-48385), 1:1000 dilution for immunoblotting and 1:200 for immunostaining.<br>anti-nucleolin antibody produced in rabbit (Sigma, N2662), 1:500 dilution for immunostaining.<br>anti-SPIN1 rabbit polyclonal antibody (Proteintech, 12105-1 AP), 1:500 dilution for immunoblotting.<br>anti-C11orf84 rabbit polyclonal antibody (Sigma, HPA040128), 1: 1000 dilution for immunoblotting.<br>anti-Histone H3 (trimethyl-K4) antibody (ab8580).<br>rabbit anti-Histone H3(trimethyl-K9) antibody (Abcam, ab8898).<br>anti-HP1γ, clone 42s2 antibody (Millipore, 05-690).
Validation	All antibodies used in this study were validated by the manufacturers for specific detection of the antigen and species reactivity and application.

Eukaryotic cell lines

Policy information about cell lines

Cell line source(s)	HEK293T/17 ATCC, Cal# CRL-11268<br>U2OS cell line ATCC, Cal# HTB-96

Authentication	Cell lines were authenticated by the provider.
Mycoplasma contamination	The cells lines were regularly checked for Mycoplasma contamination. Two cell lines used in this study were Mycoplasma free.
Commonly misidentified lines<br>(See ICLAC register)	No commonly misidentified cell lines were used.
